# Draft Genome Sequences of Five Spore-Forming Food Isolates of Bacillus pumilus

**DOI:** 10.1128/genomeA.01539-14

**Published:** 2015-03-12

**Authors:** Anne de Jong, Auke J. van Heel, Manuel Montalban-Lopez, Antonina O. Krawczyk, Erwin M. Berendsen, Marjon Wells-Bennik, Oscar P. Kuipers

**Affiliations:** a Molecular Genetics, University of Groningen, Groningen, the Netherlands; b NIZO food research, Ede, the Netherlands; c Top Institute Food and Nutrition (TIFN), Wageningen, the Netherlands

## Abstract

Here, we report the draft genome sequences of five food isolates of Bacillus pumilus, a spore-forming Gram-positive bacterium.

## GENOME ANNOUNCEMENT

Bacillus pumilus is a Gram-positive rod-shaped spore-forming soil bacterium. B. pumilus and its spores are commonly resistant to extreme environmental conditions (1, 2). Therefore, B. pumilus contamination in industrial settings can be persistent. B. pumilus is also used beneficially in the production of industrially relevant compounds, such as xylanases (3), lipases (4), and proteases (5).

The five B. pumilus strains used in this study were grown overnight in 10 ml of brain heart infusion (BHI) broth (Difco) at 37°C and were harvested at the exponential growth phase after reinoculation. Following centrifugation, the cell pellet was resuspended in SET buffer (75 mM NaCl, 25 mM EDTA, 20 mM Tris-HCl [pH 7.5]) and incubated with lysozyme (2 mg/ml) and RNase (0.4 mg/ml) for 30 min at 37°C. Subsequently, the sample was treated with SDS (1% final concentration) and proteinase K (0.5 mg/ml) at 55°C for 60 min. Genomic DNA was extracted from the lysate with phenol-chloroform, precipitated with isopropanol and sodium acetate (300 mM), and dissolved in Tris-EDTA (TE) buffer. The isolated DNA was sheared to 500-bp fragments in a Covaris (KBiosciences) ultrasonicator device for preparing the next-generation sequencing (NGS) library using the paired-end NEBNext Ultra DNA library prep kit for Illumina. The libraries were 101 bases paired-end sequenced on an Illumina HiSeq 2000 by multiplexing 12 samples per flow cell. Velvet (6), in combination with VelvetOptimiser (https://github.com/Victorian-Bioinformatics-Consortium/VelvetOptimiser), was used to perform a *de novo* paired-end assembly on each of the five genomes, resulting in the draft genome sequences (Table 1). Annotation of the genomes was done using the following steps: (i) the scaffolds were uploaded to the RAST server (7) and automatically annotated using the SEED-based method on this server, (ii) the resulting annotated scaffolds were mapped using CONTIGuator (8) to their closest neighbor (as identified by RAST) to generate the pseudogenome, (iii) locus tags were added to each feature using an in-house-developed perl script, according to the NCBI standard, (iv) BAGEL3 (9) was used to find and annotate bacteriocin gene clusters, and (v) the protein annotation was extended using InterProScan (10)].

**TABLE 1:** Overview of the five B. pumilus strains in NCBI BioProject PRJNA270572

Strain	Accession no.	Isolation source
R1 B4107	JXCK00000000	Reisentopf with chicken
B4127	JXCL00000000	Cereals, cereal products, breads, pasta, and pastries
B4129	JXCM00000000	Milk, dairy products, and desserts
B4133	JXCN00000000	Cereals, cereal products, breads, pasta, and pastries
B4134	JXCO00000000	Vegetables and fermented vegetable products

### Nucleotide sequence accession numbers.

The genome sequence of the five annotated strains have been deposited as whole-genome shotgun projects at DDBJ/EMBL/GenBank under the accession numbers listed in Table 1.
